# A Role for VEGFR2 Activation in Endothelial Responses Caused by Barrier Disruptive OxPAPC Concentrations

**DOI:** 10.1371/journal.pone.0030957

**Published:** 2012-01-31

**Authors:** Anna A. Birukova, Sangderk Lee, Vitaliy Starosta, Tinghuai Wu, Tiffany Ho, Jin Kim, Judith A. Berliner, Konstantin G. Birukov

**Affiliations:** 1 Section of Pulmonary and Critical Medicine, Department of Medicine, Lung Injury Center, University of Chicago, Chicago, Illinois, United States of America; 2 Departments of Pathology and Medicine, University of California Los Angeles, Los Angeles, California, United States of America; Northwestern University Feinberg School of Medicine, United States of America

## Abstract

**Introduction:**

Oxidation products of 1-palmitoyl-2-arachidonoyl-*sn*-glycero-3-phosphatidylcholine (OxPAPC) differentially modulate endothelial cell (EC) barrier function in a dose-dependent fashion. Vascular endothelial growth factor receptor-2 (VEGFR2) is involved in the OxPAPC-induced EC inflammatory activation. This study examined a role of VEGFR2 in barrier dysfunction caused by high concentrations of OxPAPC and evaluated downstream signaling mechanisms resulting from the effect of OxPAPC in EC from pulmonary and systemic circulation.

**Methods:**

EC monolayer permeability in human pulmonary artery endothelial cells (HPAEC) and human aortic endothelial cells (HAEC) was monitored by changes in transendothelial electrical resistance (TER) across EC monolayers. Actin cytoskeleton was examined by immunostaining with Texas Red labeled phalloidin. Phosphorylation of myosin light chains (MLC) and VE-Cadherin was examined by Western blot and immunofluorescence techniques. The role of VEGFR2 in OxPAPC-induced permeability and cytoskeletal arrangement were determined using siRNA-induced VEGFR2 knockdown.

**Results:**

Low OxPAPC concentrations (5–20 µg/ml) induced a barrier protective response in both HPAEC and HAEC, while high OxPAPC concentrations (50–100 µg/ml) caused a rapid increase in permeability ; actin stress fiber formation and increased MLC phosphorylation were observed as early as 30 min after treatment. VEGFR2 knockdown dramatically decreased the amount of MLC phosphorylation and stress fiber formation caused by high OxPAPC concentrations with modest effects on the amount of VE-cadherin phosphorylation at Y^731^. We present evidence that activation of Rho is involved in the OxPAPC/VEGFR2 mechanism of EC permeability induced by high OxPAPC concentrations. Knockdown of VEGFR2 did not rescue the early drop in TER but prevented further development of OxPAPC-induced barrier dysfunction.

**Conclusions:**

This study shows that VEGFR2 is involved in the delayed phase of EC barrier dysfunction caused by high OxPAPC concentrations and contributes to stress fiber formation and increased MLC phosphorylation.

## Introduction

Endothelial barrier dysfunction plays an important role in a number of chronic and acute inflammatory diseases such as atherosclerosis and lung pathologies including asthma, acute lung inflammation and its severe complication, acute respiratory distress syndrome (ARDS).

Phospholipid oxidation products, specifically oxidized 1-palmitoyl-2-arachidonoyl-*sn*-glycero-3-phosphatidylcholine (OxPAPC), derived from lipoproteins and membranes of cells undergoing oxidative stress or apoptosis, have been shown to accumulate in a number of inflammatory diseases including atherosclerosis, lung inflammation and tissue injury [1], [2]. OxPAPC has multiple effects on the vascular endothelium including a change in the expression of approximately 1500 genes [3] and the phosphorylation of 228 molecules regulating multiple pathways involved in inflammation, sterol regulation, coagulation, cell cycle and cell junctions [4]. Increased levels of oxidized phospholipids present in the injured lung may influence pulmonary endothelial cell (EC) functions including the modulation of pulmonary inflammatory response and EC barrier regulation [5], [6], [7], [8].

Previous studies demonstrated that OxPAPC concentrations in the 5–20 µg/ml range enhanced endothelial monolayer barrier properties in vitro, and similar doses of intravenously injected OxPAPC protected lung barrier function and reduced inflammation in models of acute lung injury caused by LPS injection or mechanical ventilation at high tidal volume [6], [7], [8]. Protective effects of OxPAPC involved enhancement of peripheral actin cytoskeleton, adherens junctions and tight junctions mediated by Rac and Rap1 GTPases [9], [10]. In contrast, high OxPAPC concentrations caused adverse effects on endothelial barrier function by increasing EC permeability and disrupting cell-cell junction complexes [11], [12], [13].

In order to reconcile the difference in the reported effects of OxPAPC on endothelial cells from different vascular beds and examine the mechanism by which high doses of OxPAPC increased EC permeability, we used two different endothelial cell types isolated from pulmonary and systemic circulation, which were exposed to low and high OxPAPC concentrations. Previous studies by our group in human aortic endothelial cells (HAEC) demonstrated an activation of vascular endothelial growth factor receptor-2 (VEGFR2), as measured by increased tyrosine phosphorylation at Y1175,in response to high OxPAPC concentrations. These high levels of OxPAPC led to the activation of SREBP and Erk-1,2 MAP Kinase signaling, and the expression of LDL receptor and inflammatory molecules- interleukin-8 and tissue factor [14]. There is evidence that VEGFR2 signaling may also lead to increased endothelial permeability *in vivo* and *in vitro* [15], [16], [17]. We present evidence for a role of VEGFR2 in cytoskeletal remodeling and increased EC monolayer permeability by high concentrations of OxPAPC via a Rho - Rho kinase - myosin light chain phosphorylation dependent mechanism.

## Results

### Dose-dependent effects of Ox-PAPC on permeability responses of pulmonary and aortic endothelial cell types

**Figure 1:**
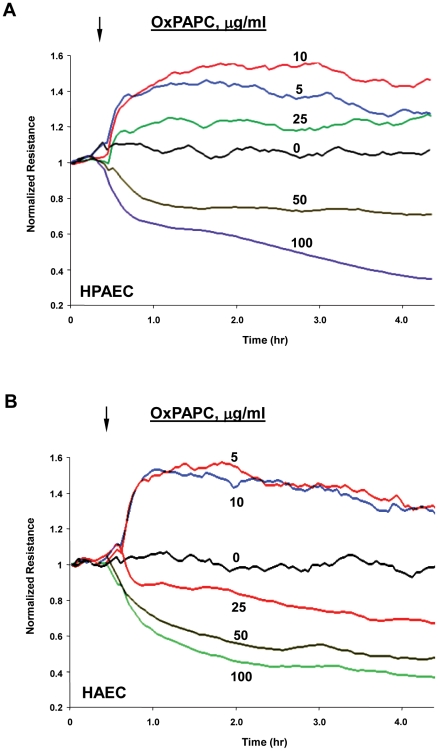
Dose dependent effects of OxPAPC on transendothelial electrical resistance of human pulmonary artery and aortic endothelial cells. **A** - Human pulmonary artery endothelial cells (HPAEC); and **B** - human aorta endothelial cells (HAEC) were seeded in polycarbonate wells with gold microelectrodes. After 24 hr of culture, HPAEC were stimulated with various OxPAPC concentrations (5, 10, 25, 50 and 100 µg/ml) or vehicle at the time indicated by arrow, and measurements of transendothelial electrical resistance (TER) were monitored over 4 hrs using an electrical cell-substrate impedance sensing system (ECIS). Results are representative of five independent experiments.

Previous reports by our group characterized dose dependent effects of OxPAPC on barrier properties of human pulmonary artery endothelial cells (HPAEC). In these experiments, we examined whether differential barrier responses to low and high OxPAPC concentrations represent a general feature of endothelial cells regardless of their origins. We compared the time course and dose response effects of OxPAPC on barrier function in HAEC and HPAEC. The maximal barrier-enhancing effect was observed in HPAEC (**Figure 1A**) and HAEC (**Figure 1B**) treated with a similar range of OxPAPC concentrations (5–10 µg/ml). Higher OxPAPC concentrations did not further enhance the EC monolayer barrier properties, but instead decreased barrier function in both HAEC and HPAEC. The most prominent barrier disruptive response was observed in the 50–100 µg/ml range of OxPAPC. The dose range that we employed for high OxPAPC in the present study was approximately one-fifth of the levels we previously demonstrated to be present in the vessel wall of hypercholesterolemic rabbits [18]. The TER increase observed at low OxPAPC concentrations developed in 5–15 min after stimulation, reached maximal levels by 30 min and lasted more than 3 hours. High OxPAPC doses caused rapid TER response, but in the opposite direction. In both HPAEC and HAEC, permeability increased and was sustained up to 4 hours when induced by high OxPAPC concentrations (50–100 µg/ml).

### Cytoskeletal remodeling induced by low and high Ox-PAPC doses in two endothelial cell types

**Figure 2:**
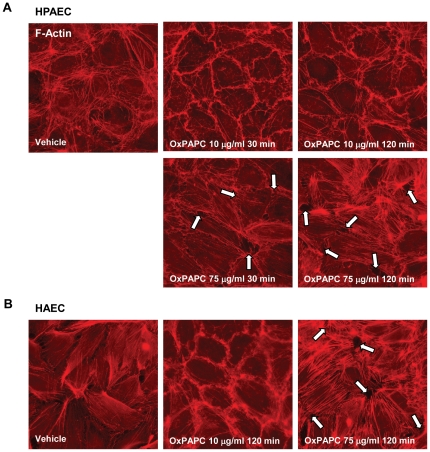
Dose dependent effects of OxPAPC on HAEC and HPAEC actin cytoskeletal remodeling and monolayer integrity. **A** – HPAEC, and **B** – HAEC monolayers grown on glass coverslips were stimulated with OxPAPC (10 µg/ml or 75 µg/ml) for 30 min or 120 min followed by immunofluorescence staining for F-actin. Arrows depict areas of intercellular gaps caused by treatment with 75 µg/ml OxPAPC in HPAEC or 50 µg/ml OxPAPC in HAEC. Shown are representative results of three independent experiments.

We next examined the effects of high and low OxPAPC doses on EC actin cytoskeletal arrangement using immunofluorescence staining of EC monolayers with Texas Red-conjugated phalloidin. Untreated HPAEC displayed generally random F-actin distribution throughout the cells with some localization of actin filament bundles at the cell boundaries (**Figure 2A**, left panel). A Similar cytoskeletal arrangement, but with more even cellular F-actin, was observed in HAEC (**Figure 2B**, left panel) Treatment with barrier enhancing OxPAPC concentration (10 µg/ml) caused redistribution of actin filaments to the cell periphery with formation of the dense F-actin rings at 30 and 120 minutes of OxPAPC stimulation in both HPAEC and HAEC (**Figure 2A,B**). In turn, barrier disruptive response to 75 µg/ml OxPAPC manifested by formation of paracellular gaps (shown by arrows) was observed in both HPAEC and HAEC (**Figure 2A,B**). Of note, the appearance of central stress fibers was slightly detectable after 30 minutes but strongly increased by 120 minutes of OxPAPC treatment (**Figure 2A**, right panels).

### Involvement of VEGFR2 in Ox-PAPC mediated decreases in barrier function

**Figure 3:**
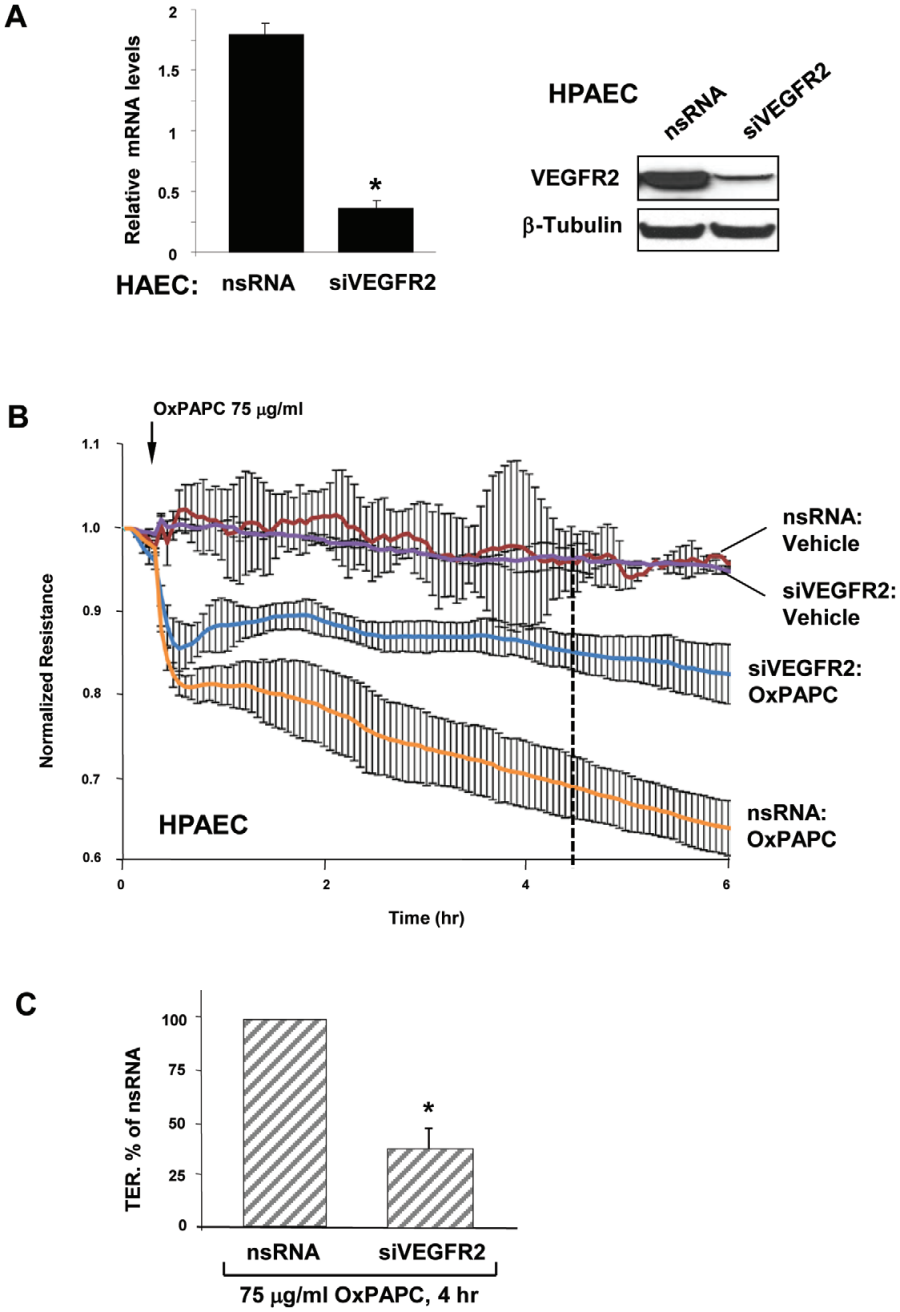
Involvement of VEGFR2 in EC barrier dysfunction induced by high OxPAPC concentration. **A** – siRNA-induced VEGFR2 depletion in HAEC and HPAEC at mRNA and protein levels was compared to treatment with non-specific RNA. Membrane re-probing with β-tubulin antibodies was used as normalization control. **B** – HPAEC grown on gold microelectrodes for TER measurements were transfected with siRNA specific to VEGFR2 (100 nM). Control cells were transfected with non-specific RNA. After 72 hrs of transfection, cells were stimulated with OxPAPC (75 µg/ml, shown by arrow) or vehicle, and permeability changes were monitored over 6 hrs. **C** - Bar graphs depict TER changes measured after 4 hrs of Ox-PAPC stimulation marked by dotted line in panel B; n = 4–6 per condition; *p<0.05.

We have previously shown that 50 µg/ml, but not 10 µg/ml OxPAPC significantly activated VEGFR2. We have also shown that VEGFR2 activation at 50 µg/ml OxPAPC leads to an increase in interleukin-8 (IL-8) and LDL receptor expression, while these effects were blocked by VEGFR2 depletion using gene-specific siRNA [14]. Our and other groups have demonstrated an important role for VEGF in regulating barrier permeability in microvascular and macrovascular EC [19], [20], [21], [22], [23]. In order to test potential involvement of VEGFR2 in decreased barrier function mediated by OxPAPC, HPAEC were transfected with siRNA to VEGFR2 or nonspecific RNA. We obtained an approximately 90% knockdown at the RNA and protein levels (**Figure 3A**). Transfection with non-specific RNA or siRNA to VEGFR2 did not affect the basal TER levels or the early phase of TER drop (before 30 min). However, the late phase of OxPAPC-induced TER decline (after 1 hour) was significantly attenuated in EC with depleted VEGFR2, as compared to cells transfected with nonspecific RNA (**Figure 3B**). These data suggest that the initial drop in TER induced by high OxPAPC dose is not mediated by VEGFR2, whereas the sustained drop requires VEGFR2 activity. The average of 6 separate TER measurements shows a strong suppression of EC monolayer barrier dysfunction observed in the EC monolayers with depleted VEGFR2 after 5 hours of high dose OxPAPC treatment (**Figure 3C**).

### Depletion of VEGFR2 attenuates stress fiber formation induced by high Ox-PAPC doses in pulmonary and aortic EC

**Figure 4:**
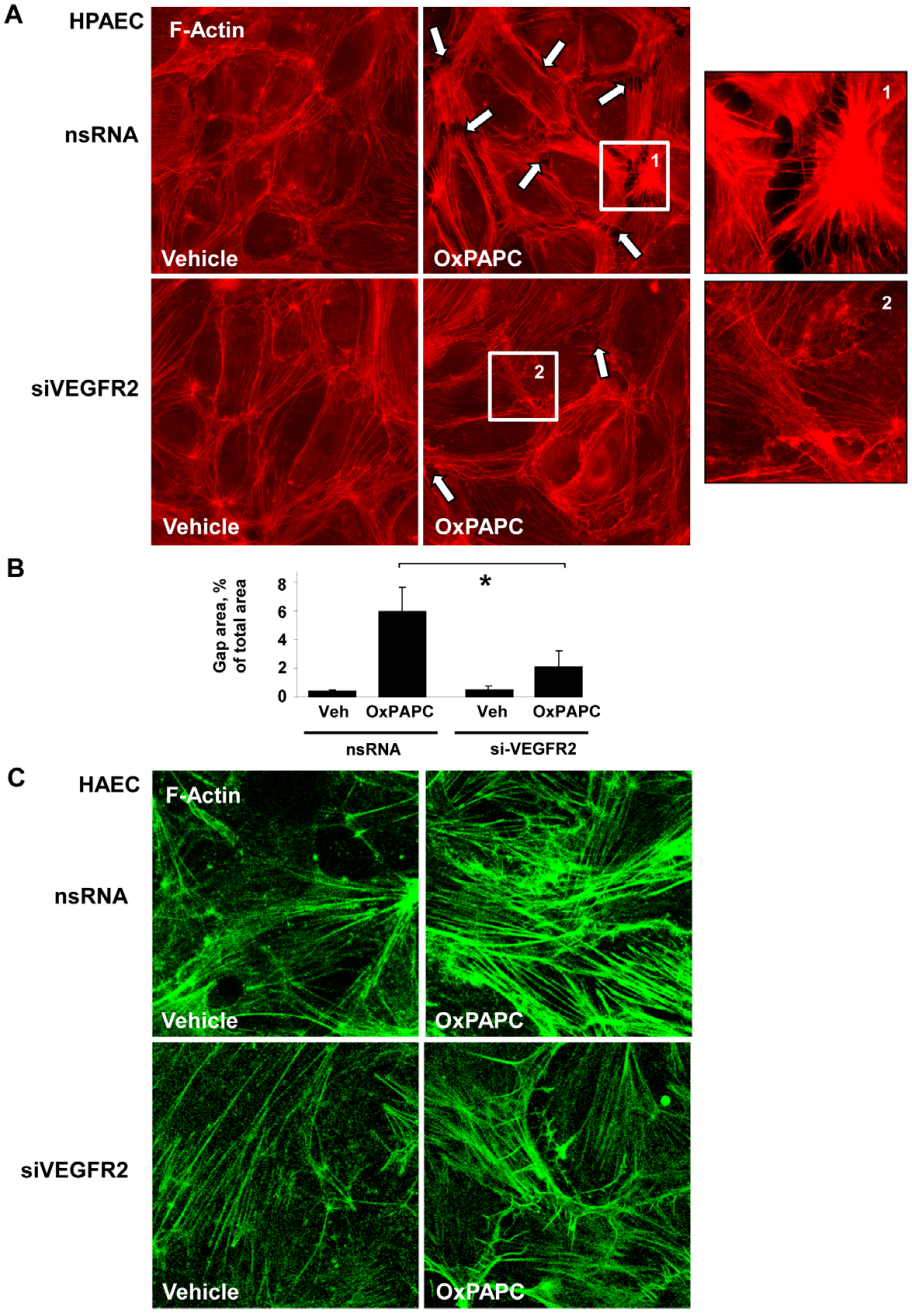
Effect of VEGFR2 depletion on actin remodeling in pulmonary EC exposed to high OxPAPC concentration. HPAECs were transfected with VEGFR2-specific siRNA. Control cells were treated with non-specific RNA. **A** - After 72 hrs of transfection cells were stimulated with OxPAPC (75 µg/ml, 120 min) or vehicle followed by immunofluorescence staining for F-actin. Paracellular gaps are marked by arrows. Insets depict higher magnification images of cell boundaries showing increased gap formation in Ox-PAPC challenged EC treated with nsRNA, which was dramatically reduced in EC treated with VEGFR2 siRNA. **B** - Quantitative analysis of gap formation in OxPAPC treated HPAEC transfected with nsRNA or VEGFR2 siRNA; n = 4–6 per condition; *p<0.05. **C** - After 72 hrs of transfection with VEGFR2-specific or non-specific RNA HAECs were stimulated with OxPAPC (50 µg/ml, 120 min) or vehicle followed by immunofluorescence staining for F-actin.

The effect of VEGFR2 depletion on F-actin remodeling was studied in HPAEC treated with high OxPAPC doses (75 µg/ml, 2 hrs). Vehicle treated cells transfected with either nsRNA or siVEGFR2 showed similar patterns of actin distribution (**Figure 4A**, left panels). High OxPAPC doses caused an increase in stress fibers and formation of paracellular gaps by 2 hours in cells transfected with nsRNA. These changes were reduced in the EC treated with VEGFR2 siRNA (**Fig. 4A** **middle and right panels**). Quantitative analysis of paracellular gap formation is presented in **Figure 4B**. A similar dramatic reduction of stress fiber formation induced by high OxPAPC doses was seen in HAEC treated with VEGFR2 siRNA (**Figure 4C**).

### Mechanism of VEGFR2 regulation of barrier function

**Figure 5:**
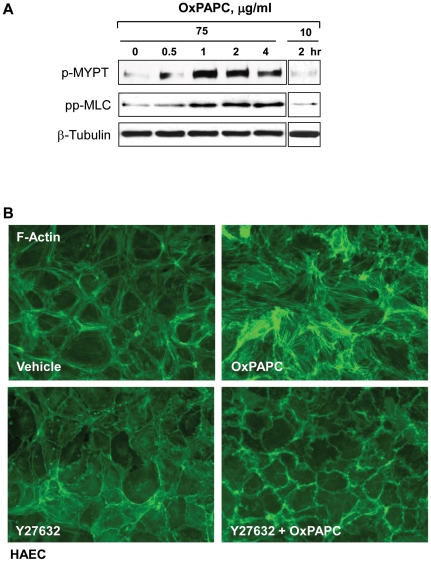
Involvement of Rho pathway in cytoskeletal effects of high OxPAPC concentrations on endothelial cells. **A** - Time course of myosin light chain phosphatase (MYPT) and myosin light chain (MLC) phosphorylation induced by OxPAPC (75 µg/ml and 10 µg/ml) was examined by western blot with corresponding phospho-specific antibodies. β-Tubulin antibody was used as normalization control. **B** - HAEC were pretreated with vehicle or Y-27632 (2 µM, 30 min) prior to stimulation with OxPAPC (50 µg/ml, 2 hrs) followed by immunofluorescence staining for F-actin.

Stress fiber formation and gap formation in EC monolayers is often associated with the activation of myosin light chain (MLC) phosphorylation, leading to the stimulation of actomyosin contractility, EC retraction, and increased EC permeability [24]. RhoA GTPase signaling plays a pivotal role in the induction of MLC phosphorylation in pulmonary endothelium via phosphorylation and inactivation of myosin phosphatase (MYPT) by Rho associated kinase [25], [26], [27]. The high dose of OxPAPC stimulated the phosphorylation of MYPT and MLC in a time-dependent manner with a significant increase observed at 30 min and a maximum response by 1 hour (**Figure 5A**). Of note, OxPAPC at barrier protective concentration (10 µg/ml) did not induce MYPT or MLC phosphorylation. To further verify the involvement of Rho signaling in stress fiber formation induced by high OxPAPC doses, we pretreated HAEC with Rho Kinase inhibitor Y-27632 prior to OxPAPC treatment. Inhibition of Rho kinase abolished OxPAPC-induced stress fiber formation (**Figure 5B**).

**Figure 6:**
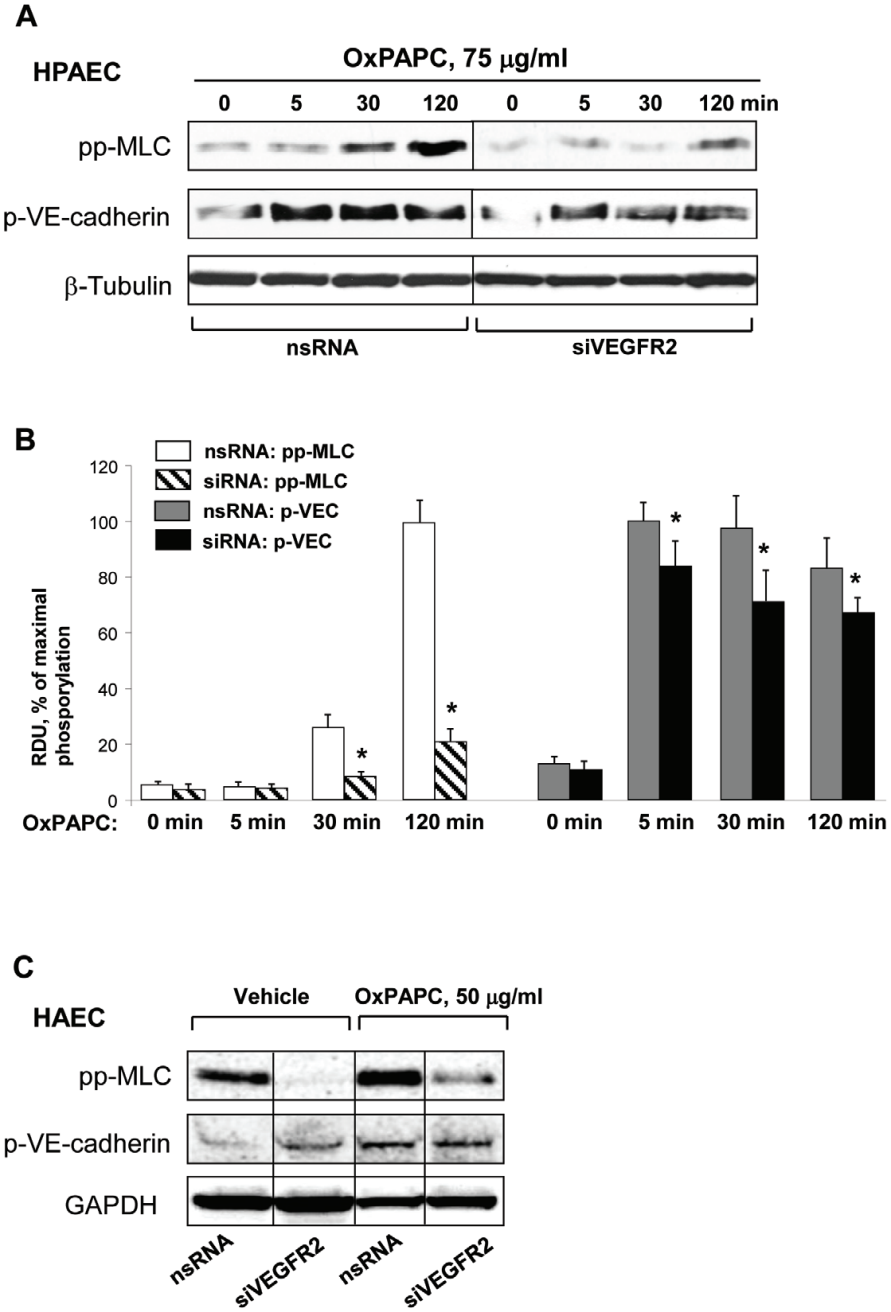
Effect of VEGFR2 depletion on phosphorylation of MLC and VE-cadherin induced by high OxPAPC concentration. HPAECs were transfected with VEGFR2-specific siRNA. Control cells were treated with non-specific RNA. After 72 hrs of transfection, cells were stimulated with OxPAPC (75 µg/ml) or vehicle. **A** – Time-dependent phosphorylation of MLC and VE-cadherin in OxPAPC-stimulated EC was detected by western blot with diphospho-MLC and phospho-Y^731^-VE-cadherin specific antibodies. Equal protein loading was confirmed by probing of membranes with β-tubulin antibodies. **B** - Quantitative analysis of MLC and VE-cadherin phosphorylation. All experiments were repeated three times. Values are mean ± SD, * *p*<0.05 vs control. **C** – Phosphorylation of MLC and VE-cadherin in HAEC treated with nonspecific and VEGFR2-specific siRNA and stimulated with OxPAPC (50 µg/ml, 30 min) was detected by western blot with diphospho-MLC and phospho-Y^731^-VE-cadherin specific antibodies. Probing with antibody to GAPDH was used as protein loading control.

Previous reports demonstrated that the activation of VEGFR2 stimulates RhoA signaling, RhoA-mediated cytoskeletal remodeling, and actomyosin contractility, leading to an increase in permeability of endothelial monolayers [16], [23], [28]. We next examined whether VEGFR2 is involved in the increase of MLC phosphorylation observed in EC treated with high OxPAPC doses. HPAEC were transfected with either VEGFR2-specific siRNA or non-specific RNA. Consistent with results shown above, treatment of HPAEC with 75 µg/ml OxPAPC did not induce rapid MLC phosphorylation (no increase at 5 min). However, MLC phosphorylation was seen at 30 and 120 minutes and was strongly inhibited by VEGFR2 depletion (**Figure 6A**). Quantitative analysis of MLC phosphorylation in control and VEGFR2 depleted HPAEC is presented in **Figure 6B**. Increased MLC phosphorylation in response to high OxPAPC dose was also observed in HAEC treated with nonspecific RNA, while depletion of VEGFR2 strongly inhibited MLC phosphorylation (**Figure 6C**).

The kinetics of the VEGFR2 effect on TER suggested that there were different regulators of the early and late decreases in barrier function caused by high dose OxPAPC and that VEGFR2 activation mainly controls the later phase. The current results and our recent published studies [29] suggest that the phosphorylation of adherens junction proteins such as VE-cadherin may be involved in the early phase of junction breakdown and that an increase in MLC phosphorylation regulates the later phase. To test the involvement of VEGFR2 in OxPAPC-induced VE-Cadherin phosphorylation, we examined the effect of knockdown of VEGFR2 on VE-Cadherin phosphorylation (**Figure 6A**). Phosphorylation of VE-Cadherin induced by high OxPAPC dose was rapid, reaching maximal levels by 5 minutes, and was only slightly attenuated by VEGFR2 knockdown in HPAEC (**Figure 6B**). In HAEC, VE-Cadherin phosphorylation was also increased by 5 minutes, and this increase was VEGFR2 independent (**Figure 6C**).

**Figure 7:**
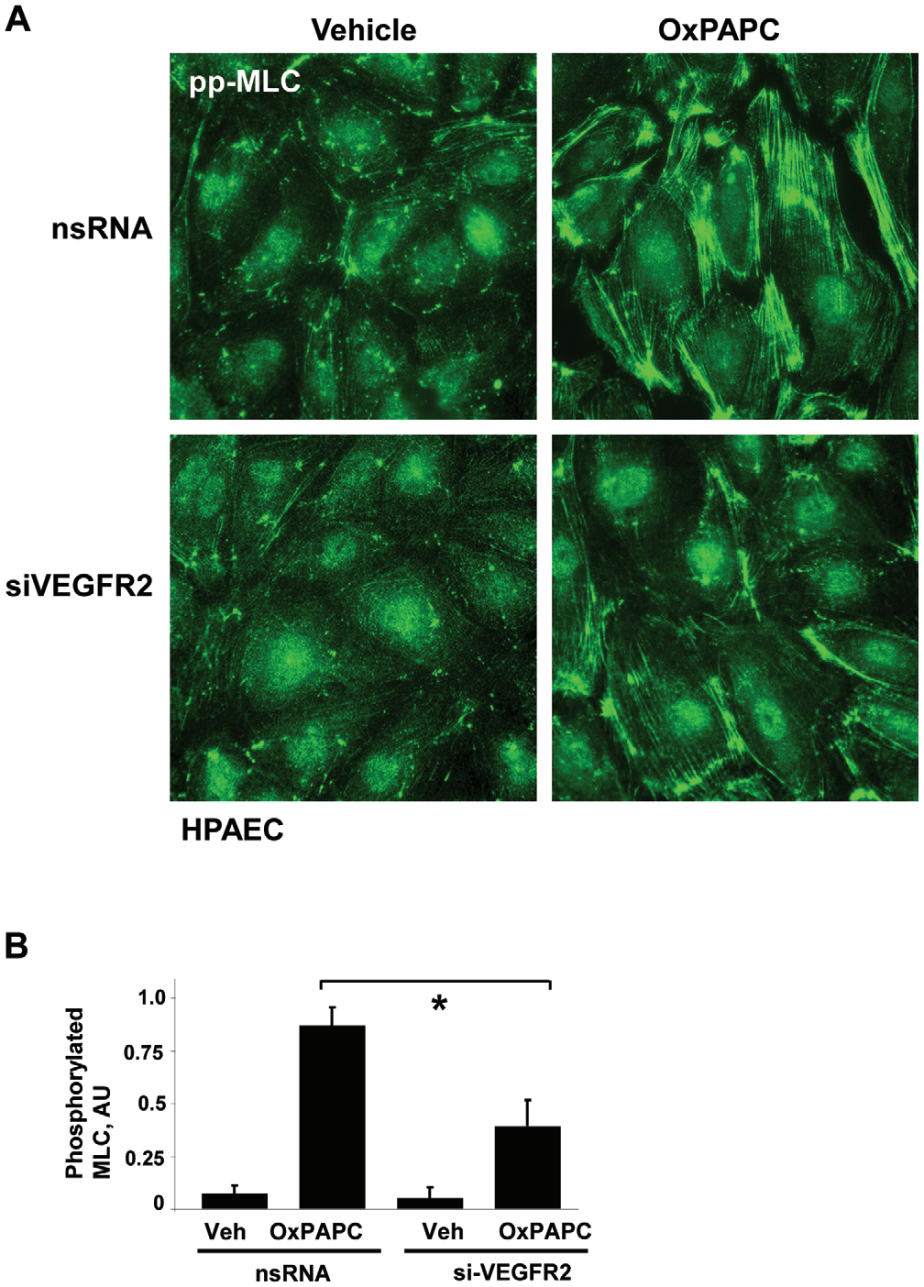
Effect of VEGFR2 depletion on intracellular localization of phophorylated MLC in HPAEC treated with high OxPAPC concentration. HPAEC transfected with VEGFR2-specific siRNA or non-specific RNA were stimulated with OxPAPC (75 µg/ml) or vehicle. **A** – Immunofluorescence staining was performed using diphospho-MLC antibodies as described in Methods. **B** - Quantitative image analysis of MLC phosphorylation was performed as described in Methods. All experiments were repeated three times. Values are mean ± SD, * *p*<0.05 vs. control.

We also examined the intracellular distribution of diphosphorylated MLC (ppMLC) by double immunofluorescence staining with diphospho-MLC specific antibody. Vehicle treated cells transfected with either nsRNA or VEGFR2 siRNA showed low levels of diphospho-MLC immunoreactivity (**Figure 7A**, left panels). High OxPAPC dose caused a strong increase in ppMLC aligned with stress fibers. In contrast to nsRNA, treatment siVEGFR2 dramatically reduced ppMLC immunoreactivity in OxPAPC-treated cells (**Figure 7A**, right panels). Quantitative analysis of MLC phosphorylation in control and VEGFR2 depleted HPAEC is presented in **Figure 7B**. These results strongly suggest that phosphorylation of MLC induced by high OxPAPC doses contributes to increased EC permeability and occurs via VEGFR2 dependent mechanism.

**Figure 8:**
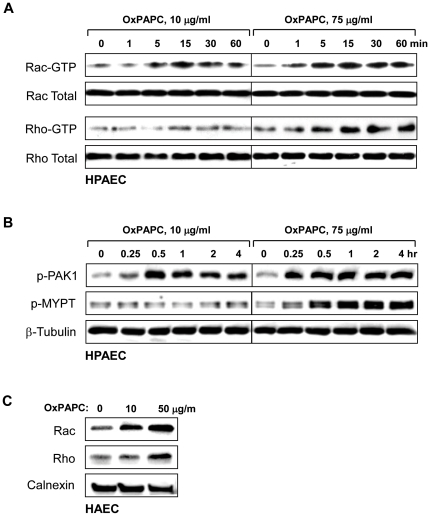
Effect of high and low OxPAPC concentrations on Rac and Rho activation. **A** - Time course of Rac and Rho activation induced by low (10 µg/ml) and high (75 µg/ml) OxPAPC concentrations was monitored using Rac- and Rho- GTP pulldown assays. Western blot analysis of Rac and Rho content in total HPAE cell lysates was used as normalization control. **B** - Time course of PAK1-Thr^423^ and MYPT1-Thr^850^ phosphorylation in HPAEC induced by low (10 µg/ml) and high (75 µg/ml) OxPAPC concentrations was monitored by western blot with corresponding phospho-specific antibodies. β-Tubulin antibody was used as normalization control. **C** – Membrane translocation of Rac and Rho after 4 hrs of HAEC stimulation with 10 µg/ml or 50 µg/ml of OxPAPC was monitored by western blot with corresponding antibodies. Calnexin antibody was used as normalization control for membrane fractions.

It remains unclear what is the mechanism of the switch that converts the OxPAPC protective effect to a disruptive effect. Our previous studies show that the protective effect of low OxPAPC doses is mediated by Rac, while Rho signaling was not activated [11]. The present results show that delayed activation of Rho signaling contributes to the EC barrier disruptive response at high OxPAPC concentrations. We next addressed the question of whether the Rac protective effect is overcome by Rho, or whether Rac is not activated by high OxPAPC concentrations. To distinguish between these alternatives, we measured Rac and Rho activation in the same EC treated with high and low OxPAPC doses for different time periods. Low OxPAPC concentrations caused early activation of Rac and autophosphorylation (at S423) of Rac effector PAK1 reflecting PAK1 activation, while Rho activity was not affected by low OxPAPC concentrations (**Figure 8AB**). High OxPAPC concentration also caused both the early Rac activation and PAK1 phosphorylation. However high OxPAPC caused a delayed activation of Rho and phosphorylation of Rho kinase target MYPT1 which was observed after 30 min (**Figure 8AB**). In agreement with these data, treatment of HAEC with low OxPAPC concentrations induced membrane translocation of Rac, but not Rho, while high OxPAPC concentrations caused membrane translocation of both Rac and Rho GTPases (**Figure 8C**). Taken together with experiments using inhibition of Rho pathway (Figure 5B), our results suggest that at high OxPAPC concentrations, the barrier protective effect of Rac activation is overcome by Rho signaling, which contributes to the late phase of EC barrier failure via activation of stress fiber formation and actomyosin contractility. In addition, pronounced and sustained Rac activation caused by high OxPAPC concentrations may stimulate NADPH oxidase activity and cause oxidative stress, which may also contribute to EC barrier compromise [30], [31]. These mechanisms will be discussed below.

## Discussion

Oxidized phospholipids play a critical role in vascular endothelial barrier regulation in health and disease. Pathologic elevation of oxidized phospholipids associated with hyperlipidemia, organ failure, or acute lung injury may compromise the vascular endothelial barrier and promote pathological processes such as atherosclerosis or lung injury [2], [32]. Previous studies demonstrated barrier protective effects of low OxPAPC concentrations on pulmonary vascular EC in culture [9], [10], [12] and in animal models of sepsis and lung injury induced by bacterial lipopolysaccharide or pathologic mechanical ventilation [1], [6], [7], [8], [33]. In contrast, high OxPAPC concentrations led to barrier dysfunction in pulmonary EC [11], [12], [13], which is associated in part with OxPAPC-induced reactive oxygen species (ROS) production and degradation of tight junction protein occludin [13].

Determination of the in vivo concentrations of OxPL under physiologic and pathologic conditions remains an open issue. Published measurements differ significantly and often represent different OxPL species measured in different body compartments. In two reports, combined concentration of all oxidized phospholipids in human plasma and tissue samples ranged from 5.4 to 51 µM [34], [35]. OxPL levels in the normal human plasma have been estimated in the range of 0.1–1.0 µM [36], but local tissue concentrations in atherosclerotic regions of the vessels have been reported in the range of 10–100 µM/kg [18], [37]. In the most recent studies, we used for the first time a “phospholipidomics” analysis by mass-spectrometry to characterize OxPL generation in the model of LPS-induced lung injury. Lungs were collected 24 and 60 hours after intratracheal LPS injection. The results demonstrated a significant elevation of OxPL products (both fragmented and oxygenated phosphatidylcholine derivates) and their presence in lung tissue samples at micromole levels (K. Birukov and V. Bochkov, unpublished data). Thus, cumulative OxPL levels in LPS-challenged lungs are consistent with 50–100 µg/ml concentrations used in this study.

Since OxPAPC accumulates in many disease settings, we thought it was important to obtain an understanding of the mechanism of its permeability regulation . This study examined whether the dose dependent effects of OxPAPC can be reproduced in EC from both systemic and pulmonary circulation. In addition, we investigated a mechanism of endothelial barrier dysfunction induced by high OxPAPC concentrations.

One major finding of this study is the demonstration of similar barrier protective effects of low OxPAPC concentrations on pulmonary and aortic EC and barrier disruptive responses to high OxPAPC doses by both cell types. However, HAEC were slightly more sensitive to the barrier disrupting effects of OxPAPC. We previously identified the signaling mechanisms by which low OxPAPC increased the endothelial barrier and demonstrated a major role of Rac GTPase-dependent strengthening of the cytoskeleton and adherens junctions [9], [38], [39], [40]. The current study focused on the effects of higher OxPAPC concentrations leading to increased EC permeability. Treatment with high concentrations of OxPAPC caused rapid barrier dysfunction (observed after 5–10 minutes). Increased permeability lasted for at least 5 hours without toxic effects on HPAEC monolayers (as tested by live/dead cell assay, data not shown). In a previous study in HAEC (using the same concentrations of OxPAPC as in the current study), survival experiments were performed using propidium iodide assay which showed a lack of toxicity [41].

High OxPAPC doses induced rapid and significant tyrosine phosphorylation of adherens junction protein VE-cadherin, which leads to VE-cadherin internalization and abrupt EC barrier dysfunction caused by the disassembly of adherens junctions [29]. In the present study, we turned our attention to the question of whether stress fiber formation and MLC phosphorylation might play a role in the late phase of barrier decrease mediated by high concentrations of OxPAPC. In contrast to enhancement of peripheral actin cytoskeletal rim and unchanged levels of phosphorylated MLC observed in EC treated with barrier protective OxPAPC concentrations [11], [42], treatment with high OxPAPC concentrations increased MLC phosphorylation by 30 minutes, which was maximal by one hour and remained elevated for at least 4 hours. The increase in stress fibers and gaps in the EC monolayers followed this time course suggesting that stress fiber formation contributed to the long-term decrease in barrier function caused by high concentrations of OxPAPC in pulmonary and aortic EC.

In search of upstream mechanisms stimulating MLC phosphorylation and stress fiber formation by high OxPAPC concentrations, we examined the potential involvement of VEGFR2. Our previous studies have demonstrated that OxPAPC induced VEGFR2 activation and examined the mechanism by which Ox-PAPC activates VEGFR2. We found that high OxPAPC doses induce high levels of Src activation [29], and activation of VEGFR2 by OxPAPC can be mostly blocked by pre-treatment with Src kinase inhibitor PP2 [14]. These data strongly suggested Src-dependent mechanism of VEGFR2 transactivation by high OxPAPC concentrations [14]. VEGFR2 activation by its canonical ligand, VEGF, leads to activation of RhoA GTPase [16], [28], [43]. The exact mechanism of VEGFR2-induced RhoA activation remains unclear, but may involve heterotrimeric protein Gq/11 and phospholipase C [43]. In turn, the activation of RhoA and Rho-associated kinase leads to the inactivation of myosin light chain phosphatase resulting in increased MLC phosphorylation, stress fiber formation and increased endothelial permeability [25]. Of note, the activation of VEGFR2 has been previously demonstrated after 5 minutes of stimulation with 40 µg/ml OxPAPC, and that activation was sustained for at least 4 hours [14].

Interestingly, we found that the rapid phase of TER decrease in the first 30 minutes of EC treatment with high OxPAPC concentrations did not involve activation of MLC phosphorylation and stress fiber formation and was not affected by VEGFR2 depletion. High OxPAPC concentrations increased VE-cadherin tyrosine phosphorylation at Y^731^ , known to be associated with EC barrier dysfunction [44], but VEGFR2 depletion caused very little changes in VE-cadherin phosphorylation state. On the other hand, TER continued to gradually decrease over a period of 1–5 hours of treatment with high OxPAPC concentration. This decrease was associated with increased stress fiber formation and MLC phosphorylation, while VEGFR2 depletion prevented further TER decline after 30 minutes of OxPAPC treatment. VEGFR2 depletion and pretreatment with Rho kinase inhibitor Y-27632 abolished stress fiber formation and MLC phosphorylation observed at later times of EC stimulation with high OxPAPC doses. Taken together, these results support the notion that VE-cadherin phosphorylation likely contributes to the early phase of EC barrier dysfunction caused by high OxPAPC concentrations, and this phase is largely independent of VEGFR2. In turn, activation of VEGFR2 – Rho - Rho kinase pathway mediates the late phase of barrier dysfunction and cytoskeletal remodeling in HPAEC and HAEC in response to 50–75 µg/ml OxPAPC.

The complexity of signaling pathways activated by different doses of oxidized phospholipids in endothelial cells still leaves some open questions to be addressed. Why is VEGFR2-dependent phase of Rho activation and MLC phosphorylation delayed and observed only after 30 min? One mechanism may involve negative Rac-Rho crosstalk, as Rac activation has been shown to be induced by OxPAPC [11], [39]. Another plausible explanation is the previously described OxPAPC-induced stimulation of cAMP signaling [41], [45], which reached peak activation at 5–15 min and declined by 30 min [42]. This increase in cAMP may suppress early Rho activation via direct and indirect effects on Rho and Rho kinase activities [46], [47], [48].

The other question is the role of Rac in the different effects of OxPAPC on endothelial barrier function. We have previously demonstrated Rac activation by both low and high concentrations of OxPAPC [11], [30]. Since Rac has been shown by multiple groups to be a barrier protective molecule, why does high dose OxPAPC open the barrier in spite of high levels of Rac activation? We think this can be attributed to: a) the role of Rac in activation of NAPDH oxidase enzymatic complex by high OxPAPC concentrations, which triggers ROS production and leads to oxidative stress and EC barrier dysfunction; or b) the increase in Rho activation that occurs in response to VEGFR2 activation and counterbalances the Rac impact in cytoskeletal mechanisms of barrier enhancement.

In summary, these studies suggest that VEGFR2 involvement in vascular endothelial barrier breakdown caused by high OxPAPC concentration is a general mechanism effective in both pulmonary and systemic circulation, which may help explain in part the reported activation of pathologic VEGFR2 signaling in both acute lung injury and atherogenesis. Beneficial effects of inhibition of VEGFR2 signaling in reduction of atherosclerosis in mice have been attributed to the reduction of neo-angiogenesis in the plaque [49]. However, VEGFR2 signaling may also contribute to a barrier dysfunction of endothelium from the large systemic vessels, as has been described in this study. Although the origin and pathogenic mechanisms resulting in high levels of oxidized phospholipids in the pulmonary circulation or in systemic arteries may be quite different, inhibition of VEGFR2 signaling may prove to be a reasonable strategy to suppress pathologic endothelial barrier dysfunction in a number of diseases where OxPAPC accumulates.

## Methods

### Reagents and cell culture

Diphospho-MLC and β-tubulin antibodies, rabbit anti-human VEGFR2 antibody, HRP-linked anti-mouse and anti-rabbit IgG were obtained from Cell Signaling (Beverly, MA). 1-Palmitoyl-2-arachidonoyl-*sn*-glycero-3-phosphorylcholine (PAPC) was obtained from Avanti Polar Lipids (Alabaster, AL). PAPC was oxidized by exposure to air for 72 hours. The extent of oxidation was measured by positive ion electrospray mass spectrometry (ESI-MS) as previously described [37]. After completion of oxidation, the phospholipids were stored at −70°C dissolved in chloroform and were used within 2 weeks of MS analysis. .All oxidized and non-oxidized phospholipid preparations were analyzed by the limulus amebocyte assay (BioWhittaker, Frederick, MD) and shown negative for endotoxin. All reagents for immunofluorescence were purchased from Molecular Probes (Eugene, OR). Human lung pulmonary artery endothelial cells (HPAEC) were obtained from Lonza (Walkersville, MD), cultured according to the manufacturer's protocol, and used at passages 5–7. Unless specified, biochemical reagents were obtained from Sigma (St. Louis, MO).

### Isolation and propagation of HAEC

Human aortic endothelial cells (HAECs) were isolated as described previously [50] and cultured in M199 medium supplemented with FBS (20% v/v), penicillin (100 U/mL), streptomycin (100 µg/mL), sodium pyruvate (1 mmol/L), heparin (90 µg/mL), and endothelial cell growth supplement (20 µg/mL).

### VEGFR2 depletion using siRNA approach

Two sets of VEGFR2-specific oligonucleotides (Stealth Select) were obtained from Invitrogen (Carlsbad, CA) and characterized in our previous study [14]. A GC% matched negative control siRNA (scrambled) was also obtained from Invitrogen. Transfection of EC with siRNA was performed as described previously [11], [51]. After 72 hr of transfection cells were used for experiments or harvested for western blot verification of specific protein depletion.

### Measurement of transendothelial electrical resistance

The cellular barrier properties were analyzed by measurements of transendothelial electrical resistance (TER) across confluent endothelial monolayers using an electrical cell-substrate impedance sensing system (Applied Biophysics, Troy, NY) as previously described [11], [12].

### Immunofluorescence staining

Endothelial cells were grown to confluence, stimulated with agonist of interest, and immunofluorescence staining for F-actin was performed as described elsewhere [25]. Likewise, after 72 hours of transfection with nsRNA or VEGFR2 siRNA, EC were stimulated with Ox-PAPC followed by immunofluorescence staining for F-actin using Texas Red-conjugated phalloidin or visualization of diphosphorylated MLC using phosphospecific antibody [25]. Images were processed with Adobe Photoshop 7.0 (Adobe Systems, San Jose, CA) software. Quantitative analysis of paracellular gap formation in OxPAPC treated cells transfected with nsRNA or VEGFR2 specific siRNA was performed as previously described [25], [52], [53].

### Western Blot

Protein extracts were subjected to SDS-polyacrylamide gel electrophoresis, transferred to nitrocellulose membrane, and probed with antibodies of interest, as previously described [54], [55], [56].

### GTPase activation assays

GTPase activation assays were performed using commercially available assay kits purchased from Upstate Biotechnology (Billerica, MA). In brief, after stimulation, cell lysates were collected, and GTP-bound Rac or Rho were captured using pull-down assays with immobilized PAK1-PBD or rhotekin-RBD, respectively, according to the manufacturer protocol. The levels of activated small GTPases as well as total Rac or Rho content were evaluated by western blot analysis.

### Rac and Rho membrane translocation

Cells were resuspended in buffer (10 mM HEPES, 40 mM KCl, 5 mM MgCl2, 1 mM, EDTA) containing protease and phosphatase inhibitor cocktails, and PMSF (1 mM). Cells were disrupted by 10 passages through a 26 gauge needle, followed by freezing and thawing. Lysates were centrifuged for 10 min at 4000 rpm. The supernatant was centrifuged for 1 hr 15 min at 35,000 rpm. The final membrane pellets were resuspended in RAL buffer (50 mM Tris-HCl, pH 7.5, 200 mM NaCl, 2.5 mM MgCl_2_, 1% NP-40, 10% glycerol) containing protease and phosphatase inhibitor cocktails, and PMSF (1 mM).

### Statistical analysis

Results are presented as mean ± SD of three to six independent experiments. Stimulated samples were compared to controls by unpaired Student's t-test. For multiple-group comparisons, a one-way analysis of variance (ANOVA), followed by the post hoc Tukey test, were used. P<0.05 was considered statistically significant.
